# Magnitude and factors associated with adherence to Iron-folic acid supplementation among pregnant women in Eritrean refugee camps, northern Ethiopia

**DOI:** 10.1186/s12884-018-1716-2

**Published:** 2018-04-05

**Authors:** Mekdemariam Getachew, Mebrahtu Abay, Hiwet Zelalem, Tirhas Gebremedhin, Teklit Grum, Alemayehu Bayray

**Affiliations:** 1 Department of Public Health, Aksum University, Aksum, Ethiopia; 2 Department of Public Health, College of Health Sciences, Aksum University, P.O. Box: 298, Aksum, Ethiopia; 3 Department of Public Health, College of Health Sciences, Arsi University, Assela, Ethiopia; 4 School of Public Health, College of Health Sciences, Mekelle University, Mekelle, Ethiopia

**Keywords:** Adherence, Iron-folic acid supplementation, Anemia, Pregnant women, Eritrean refugee camps, Ethiopia

## Abstract

**Background:**

Globally, anemia is a public health problem affecting the life of more than two billion people. Pregnant women are at high risk of iron deficiency anemia due to increased nutrient requirement during pregnancy. Iron-folic acid supplementation is the main strategy for prevention and control of iron deficiency anemia and its effectiveness depends on adherence to Iron-Folic Acid tablets. In the refugee camps of Ethiopia, despite the efforts made to reduce iron deficiency anemia during pregnancy, information about adherence to iron-folic acid supplementation and its associated factors are lacking. The objective of this study was to assess magnitude and factors associated with adherence to iron-folic acid supplementation, among pregnant women, in Shire refugee camps.

**Methods:**

Institution based cross-sectional study with mixed design (quantitative and qualitative) was carried out among pregnant women in Shire refugee camps from September to November 2015. For quantitative data, a sample of 320 pregnant women was systematically selected and data were collected via interview administered structured questionnaire. Quantitative data were coded and entered into Epi-info version 3.5.1 and exported into a statistical package for social sciences (SPSS) Version 19.0 software for analysis. Bivariable and multivariable logistic regressions were employed to identify the predictors at *p*-value < 0.2 and 0.05 respectively. For the qualitative part, six focus group discussions and three key informant interviews were conducted on purposely-selected individuals. Open-Code version 3.6.2.0 was used for analysis. Identified themes were arranged into coherent groupings and triangulated with quantitative findings.

**Results:**

The adherence rate was found to be 64.7% [95% CI (59.7%, 70.0%)]. Women who were having lower knowledge about anemia [AOR; 0.23 95% CI (0.14, 0.38)] and not receiving information about importance of iron-folic acid supplementation [AOR; 0.43 95% CI (0.25, 0.74)] were negatively associated with adherence to iron and folic acid., Having four or more antenatal care visits [AOR; 2.83 95% CI (1.46, 5.48)] were positively significantly associated with adherence to iron-folic acid supplementation.

**Conclusions:**

Adherence rate of iron-folic acid supplementation during pregnancy in the study area is relatively low. Proper counseling and health promotion about Iron-Folic Acid tablet intake, promoting the benefits of early and frequent ANC visit, health promotion on anemia prevention and health benefits of the importance of iron-folic acid supplements are recommended to increase adherence with iron-folic acid supplementation.

**Electronic supplementary material:**

The online version of this article (10.1186/s12884-018-1716-2) contains supplementary material, which is available to authorized users.

## Background

Globally, almost 41.8% of pregnant women and 30.2% non-pregnant women are anemic [1, 2]. In addition, 20% of maternal mortality in Sub-Saharan Africa is directly attributable to anemia [1]. In Ethiopia, 17% of reproductive age group women and 22% of pregnant women are anemic. When we look at the residence difference the magnitude of anemia among pregnant women who reside in rural and urban areas is 18% and 11%, respectively [3].

In several low-income countries, the cause of anemia during pregnancy is multi-factorial. It includes nutritional deficiencies of iron, folic acid, vitamin B-12 and parasitic diseases such as malaria and hookworm. In sub-Saharan Africa, iron and folic acid deficiencies are the most common causes of anemia among pregnant women [4].

Iron deficiency anemia contributes to adverse effects on maternal and child health. The maternal consequences of anemia, which include, but not limited to, low weight gain, preterm labor, placenta previa, premature rupture of membrane, cardiac arrest, hemorrhage, lowered resistance to infection, poor cognitive development, and reduced work capacity. Similarly, anemia during pregnancy has fetal and neonatal risks, which include prematurity, low birth weight, and fetal distress, which contribute to perinatal morbidity and mortality. Infants born to anemic women are more likely to become anemic themselves [5, 6]. Folic acid deficiency at conception and in early pregnancy is also associated with increased risk of neural tube defect, preeclampsia, fetal malformations and preterm delivery [7, 8].

In Ethiopia, iron-folic acid supplementation (IFA) is one of the main strategies for prevention of anemia. The Ethiopian national guideline for prevention of micronutrient deficiencies highlights the need for daily IFA supplementation for at least 6 months during pregnancy and 3 months postpartum [9]. But, effectiveness and success of such interventions depend on the adherence to IFA tablets. Adherence describes the degree to which a patient correctly follows a medical advice. Many experts believe that one of the main reasons that national iron supplementation programs have failed is women’s “non-compliance” [10, 11].

In spite of the WHO recommendation, the use of iron and folic acid supplementation is still low in many countries [12]. According to the Ethiopia Demographic Health Survey (EDHS) of 2011, less than 1% of pregnant women took IFA supplement for recommended period during their pregnancy; but this prevalence might not be exactly comparable with the aim of this study, which is conducted among pregnant women with full availability of IFA, as availability is a pre-requisite for utilization [3]. A study in four major regions of Ethiopia (Tigray, Amhara, Oromiya, and Southern Nations Nationalities and Peoples shows fewer than 3.5% took the supplements for more than 90 days [13].

The aim of this study was, therefore, identifying the magnitude of anemia and its determinants among pregnant women in Shire refugee camps, Ethiopia.

## Methods

### Study setting

Health facility-based cross-sectional study was carried out in four Shire refugee camps, named as “*Shimelba”, “May-Aini”, “Adi-Harush”,* and*“Hitsats”,* located in the Tigray regional state, Northern Ethiopia whereby Eritrean refugees are residing in. According to the UNHCR monthly population data, the Eritrean refugee population in the four camps was 60,119 as of 31 December 2013, of which 28.1% were females.

Data were collected from 03, November to 29, December 2015, and 1036 pregnant women were residing during the data collection period in all refugee camps.

Each refugee camp had one health center, which is administered by the Administration for Refugees and Returnees Affairs (ARRA). Comprehensive health services and nutrition programs are running in the four camps supported by UNHCR and WFP. Maternal health services have been implemented in each refugee camp. Pregnant women who came to the health centers had an access to all components of ANC services including IFA supplementation by health social workers regularly.

### Population and sampling

The source population was pregnant women residing in all of the four Shire refugee camps. The pregnant women visiting Shire refugee camps’ health centers at least for a second antenatal visit and received IFA tablets for at least one month prior to the date of interview were selected as the study population. Women who were critically ill during the data collection period were excluded from the study. The interview was made just before receiving the ANC service.

#### In quantitative part

**Fig. 1:**
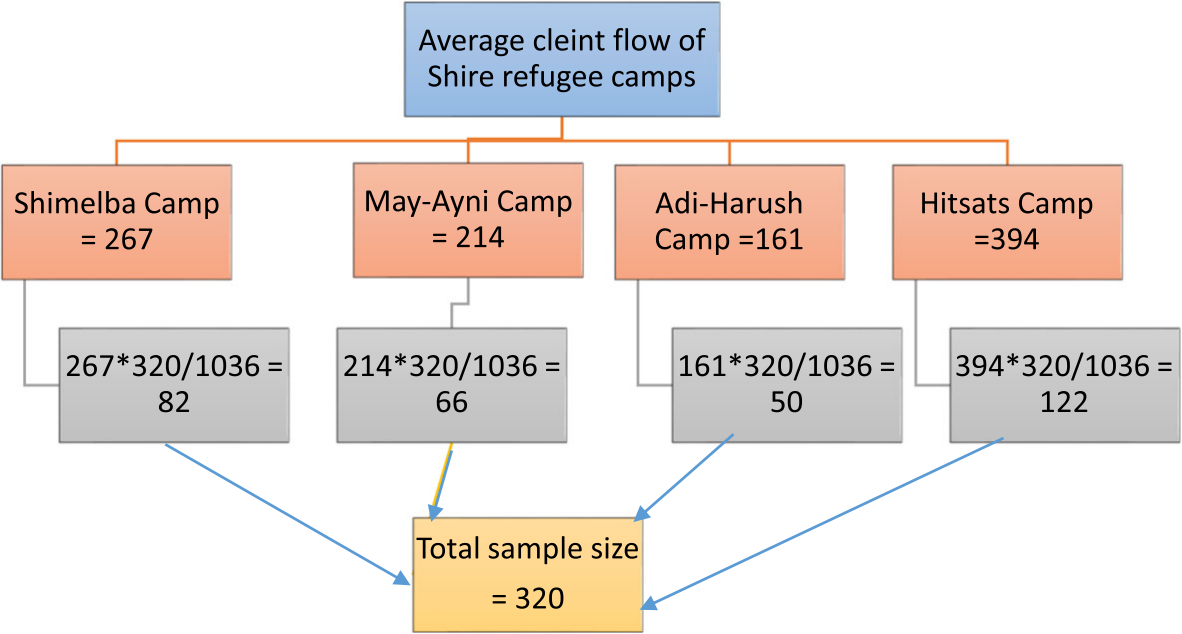
Sampling procedure for the quantitative data among pregnant women in Eritrean refugee camps, northern Ethiopia

A total of 320 pregnant women were included in the study. The sample size was calculated using a single population proportion formula with the proportion of adherence (*P* = 37%) from a study conducted in North West Tigray [14]. During the sample size calculation, the assumptions of 95% confidence interval, 5% of marginal error and 10% non-response rate were considered. To determine the number of women to be interviewed, an average of 12 months’ flow of pregnant women in each camp was obtained from UNHCR and ARRA Health Information System (HIS) report. The average number of pregnant women attending second ANC visit in each health center was; 267 in “*Shimelba”, 214 in “May-Aini”,* 161 in *“Adi-Harush”* and 394 in *“Hitsats”*. Considering the client flow in the health center, the total sample size determined (320) was allocated proportionally to each health center in the camps (Fig. 1). The final sample from each camp was done consecutively until we reach the required sample.

#### In qualitative part

A total of six Focus Group Discussions (FGDs) among pregnant women and three Key Informant Interviews (KIIs) among midwives who give ANC service for the pregnant women in the four refugee camps were conducted using purposive sampling technique until enough information is gained. A total of 56 participants were involved in the six FGDs. The average number of participants in the FGDs was 10 individuals with a range of 8 to 12 individuals. One to two FGDs were conducted in each of the four refugee camps, and pregnant women were selected purposefully based on their length of pregnancy period. To get sufficient information pregnant women with longer duration of pregnancy, usually in their third trimester, were selected.

### Data collection and quality assurance procedures

#### For the quantitative part

Structured questionnaires were used for the interview. The questionnaire was adopted and modified from Ethiopian Health and Nutrition Research Institute, micronutrient initiative effective modalities to improve pregnant women’s adherence to daily iron supplementation survey questionnaire, WHO guideline for anemia prevention and other literature [15, 16]. First, the questionnaire was written in English and then translated to the local language, Tigrigna, peer-reviewed and translated back to English to ensure consistency*.* The Tigrigna version of the questionnaire was pretested among 16 women (5% of the total sample size) who visit May-Tsebri government health center Maternal and Child Health (MCH) clinic. The principal investigator and data collectors undertook the pretest. Based on the findings of the pretest, necessary corrections and amendments were made on the questionnaire before the actual data collection was started. Four refugee diploma holder midwife data collectors (one for each camp) and two supervisors (one for two camps) who have a Bachelor of Science in nursing were recruited. Supervisors and data collectors had previous experience of data collection and were trained for one day **(**See Additional file 1 for details of the quantitative data collection tool).

#### For the qualitative part

FGD interview guide note was used to understand the perceptions of participants about the use of antenatal IFA supplementation. Furthermore, the behavioral and cultural issues in terms of facilitating factors and barriers to utilization of antenatal IFA supplementation were assessed through KII guiding questions. First, the FGD guiding questions were written in English and then translated to the local language, Tigrigna, and peer-reviewed and then translated back to English before administration. Two bachelor degree holders in nursing and sociology with experiences in collecting qualitative data were assigned in each FGD to collect data as modulator and note taker. A tape recorder was used to capture the qualitative data during FGD and KII. Key informant interview was administered by the principal investigator and one bachelor holder in nursing was a note taker (see Additional file 2 for details of the qualitative data collection tool).

### Variables and measurement

The dependent variable is adherence to iron-folic acid supplementation and the independent variables are socio-demographic, obstetric, personal/individual and health service related factors of the pregnant women.

#### Adherence to IFA

Pregnant women who reported that they took at least 70% of the expected dose of the IFA tablets in the previous two weeks before the data collection, which is equivalent to consuming at least five tablets per week, were considered as adhered to IFA supplementation, and the opposite is true for non-adherence [15, 17, 18]. All women had given sufficient IFA tablets during their first and second ANC visit. Regarding the monthly income of the respondents, a tertile classification is applied to categorize it into three levels, as low, medium and higher income categories.

#### Knowledge about anemia

To assess the level of knowledge about anemia during pregnancy, respondents were asked 18 questions on major causes, symptoms, and consequences during pregnancy. Those who scored > the mean value were considered as somehow knowledgeable and those who scored ≤the mean value were considered as less knowledgeable.

### Data management and analyses procedures

#### For the quantitative part

Data were coded and entered into Epi-info version 3.5.1 and exported into statistical package for social sciences (SPSS) Version 21.0 software for analyses. Descriptive statistics were employed to describe the study population in relation to socio-demographic and other relevant variables and to display findings in mean, median, percentage and 95% confidence intervals. A composite scale was constructed for maternal knowledge about anemia. To identify factors associated with adherence to IFA supplementation, first a bivariable logistic regression was performed for each independent variable with adherence to IFA supplementation and crude odds ratio with 95% confidence intervals was obtained. Subsequently, significant variables in the bivariable analysis (*p*-value < 0.2) were incorporated into the multivariable logistic regression to determine independent predictors of adherence to IFA supplementation among pregnant women. To control the effect of the four different health facilities (camps), a random effect was estimated using Generalized Estimating Equation (GEE) model. Results were summarized using tables, figures and graphs. The goodness of fit of the final logistic model was tested using Hosmer and Lemeshow test. For the knowledge-related questions, reliability was checked during pretest using Cronbach’s Alpha, which was 0.746.

#### For the qualitative part

Open code version 3.6.2.0 software was used for analysis. Data were recorded by tape recorders from six FGDs and three KIIs were transcribed word-by-word into plain text and translated into English. After codes were developed, issues discussed and themes developed. The codes developed from the texts were coded into the following terms: availability of tablets, knowledge about IFA supplementation, attitude about IFAs, information from health professionals, and the perception of women, as they are healthy, parity and gravidity. The themes developed from the codes were personal factors, health facility-related factors, and pregnancy-related factors. All authors together develop the codes and themes of the qualitative data. An MSc expert in Health Education assisted how to analyze the qualitative data. Finally, the themes were triangulated with quantitative findings.

## Results

### Demographic and socioeconomic characteristics of respondents

A total of 320 pregnant women were interviewed with a 100% response rate. The mean age of the study subjects was 26.7 (SD = 5.95) years. A majority, 257 (80.3%) of the respondents was between 20 and 34 years of age. More than half, 193 (60.3%) of the respondents were Orthodox Christian by religion. A significant number of respondents, 65(20.3%), were unable to read and write. Regarding their occupational status, 255 (79.7%) were housewives. In this study 199(62.2%) of respondents had an average household monthly income of above 900 Ethiopian Birr (> 40.7 USD) (Table 1).

**Table 1:** Socio-demographic characteristics of pregnant women attending antenatal clinics in Shire refugee camps, Tigray, Ethiopia, 2015. (*n* = 320)

Variables		Number	Percent
Age in years	15–19	25	7.8
	20–34	257	80.3
	> 34	38	11.9
Religion	Orthodox	193	60.3
	Muslim	56	17.5
	Catholic	49	15.3
	Protestant	22	6.9
Marital status	Single	29	9.1
	Married	283	88.4
	Divorced	8	2.5
Maternal occupation	House wife	255	79.7
	Merchant	21	6.6
	Volunteer	21	6.6
	Others^*^	23	7.2
Maternal education	Unable to read and write	65	20.3
	Primary education completed	163	50.9
	Secondary and above	92	28.8
Husband education	Unable to read and write	25	8.8
	Completed primary education	114	40.3
	Secondary and above	144	50.9
Family size	1–3	146	45.6
	4–6	142	44.4
	> 6	32	10.0
Monthly income in Ethiopian Birr (US Dollar)	< 500 (≤ 22.6)	54	16.9
	500–900 (22.6–40.7)	67	20.9
	> 900 (> 40.7)	199	62.2

Others*: daily laborer, farmer

### Obstetric and health service related characteristics of respondents

The mean gestational age of the pregnant women was 30.8(SD = ±4.4) weeks. The majority of the participants were in their third trimester 205 (64.1%). The mean gestational age of pregnant women started taking IFA supplement were at 17.1 (SD = ± 4.4) week. More than half of the women were gravida 2–4 [183 (57.2%)] and para-1 [159 (55.9%)]. Only 32 (10%) of the pregnant women had known medical illness at the time of interview (Table 2).

**Table 2:** Obstetric and health service related characteristics of pregnant women attending antenatal clinics in Shire refugee camps, Tigray, Ethiopia, 2015. (*n* = 320)

Variables		Number	Percent
Gravida (number of pregnancies)	1	78	24.4
	2–4	183	57.2
	> = 5	59	18.4
Parity (number of deliveries)	0	78	24.4
	1	171	31.5
	2–4	116	53.4
	> = 5	25	7.8
Birth order	1	179	55.9
	2–4	116	36.3
	> = 5	25	7.8
Pregnancy Trimester during interview	Second trimester	115	35.9
	Third trimester	205	64.1
Gestational Age where ANC started	Before 16 weeks	154	48.1
	At or After 16 weeks	166	51.9
Number of ANC visits	2–3	232	72.5
	≥ 4	88	27.5
Receiving information about the importance of IFA supplementation	No	139	43.4
	Yes	181	56.6
Medical illness	No	288	90.0
	Yes	32	10.0
Morning sickness	No	255	79.7
	Yes	65	20.3
Side effects	No	234	83.1
	Yes	86	26.9

### Adherence to IFA supplementation of the respondents

Adherence level was determined by self-report questions among 320 women who were given iron-folic acid supplements during pregnancy. Only 207 [64.7%; 95% CI (59.7, 70.0)] women took ≥70% of expected doses of the IFA supplements, that is, for greater than five days in a week. The primary underlying reasons for the non-adherence of IFA among the pregnant women were, occurrence of side effects [86 (40%)], unaware of the importance of IFA [65 (30.2%)], forgetfulness [31 (14.4%)], inadequate or lack of IFA in health facility [26 (12.1%)] and fear of side effects [7 (3.3%)].

This was supported by the FGD. Most of FGD participants reported that forgetfulness and drug side effects are the main reasons for non- adherence. “*I want to take the IFA supplementation as prescribed, but I repeatedly forget to take the tablet regularly.” (****A 27 years old, pregnant woman participated in FGD).*** *“All drugs, including the drugs used to prevent anemia, are very important, but the problem is that it is very difficult to take them continuously, as they have their own side effect; otherwise I believe that they are very important to our health.”* **(A 25 years old pregnant woman participated in the FGD).**

### Knowledge of respondents about anemia during pregnancy

Of the total participants, 261 (81.6%) had ever heard of anemia during pregnancy. Comprehensive knowledge of anemia was computed by summing up all relevant 18 knowledge items. The mean score was 7.7 (SD = 4.23). Accordingly, 61.3% of the respondents were somehow knowledgeable about anemia. This was supported by the FGD; most of the pregnant women mentioned fatigue, dizziness, swelling in the extremities as major sign and symptom. The risk of maternal death during labor and fetal growth retardation are also reported as consequences of anemia during pregnancy.

*“I myself have anemia. I feel dizzy and weak.”* ***(A 35-year old pregnant women having five children participated in FGD).***

### Factors associated with adherence to IFA supplementation

In the bivariable analysis, seven factors were found to be significantly associated with adherence to IFA supplementation. Maternal age [COR = 2.39, 95% CI = (0.87–6.56)], merchants [COR = 1.88, 95% CI = (1.29–2.61)], farmers/daily laborers [COR = 2.69, 95% CI (1.13–6.40)] number of ANC visits [COR = 0.31, 95% CI = (0.17–0.56)], time of ANC visit in trimester [COR = 0.47, 95% CI = (0.30–0.76)], reported morning sickness [COR = 0.65, 95%, CI = (0.35–1.18)], receiving information about importance of IFA supplementation [COR = 0.29, 95% CI = (0.18–0.47)] and maternal knowledge about anemia [COR = 0.25, 95% CI = (0.14–0.43)] at *P*-value of < 0.2. Ultimately, frequency of ANC visit, receiving information about the importance of iron supplementation and maternal knowledge about anemia were identified as significant independent predictors in the multivariable logistic regression analysis after adjusting to each other at (*P*-value < 0.05).

Therefore, controlling all other variables, having less knowledge about anemia during pregnancy was negatively associated with adherence to IFA supplementation **[AOR = 0.25, 95% CI = (0.14–0.43)].** Not receiving information about the importance of IFA supplementation during pregnancy was negatively associated with adherence to IFA **[AOR = 0.43, 95% CI = (0.25–0.74)].** ANC visit of four times or more was positively related to adherence to IFA supplement **[AOR = 2.83, 95% CI = (1.46–5.48)]** (Table 3).

**Table 3:** Factors associated with adherence to IFA supplementation among pregnant women attending antenatal clinics in Shire refugee camps, Tigray, Ethiopia, 2015. (*n* = 320)

Variables		Adherence for IFA supplementation		COR (CI 95%)	AOR (CI 95%)
		Yes	No		
Age	15–19	20	5	1.00	1.00
	20–34	161	96	2.39(0.87–6.56)	3.09(1.02–9.40)
	> 35	26	12	1.85(0.56–6.10)	2.60(0.68–9.88)
Maternal occupation	House wife	172	83	1.00	1.00
	Merchant	11	10	1.88(0.77–0.61)	0.83(0.29–2.36)
	Volunteer	14	7	1.04(0.40–2.66)	0.76(0.27–2.15)
	Others^a^	10	13	2.69(1.13–6.40)	0.59(0.22–1.58)
Time of ANC started	< 16 weeks of GA	41	113	1.00	1.00
	≥16 weeks of GA	72	94	0.47(0.30–0.76)	0.87(0.50–1.50)
Number of ANC visit	< 4 visits	97	135	1.00	1.00
	≥4 visits	16	72	0.31(0.17–0.56)	**2.83(1.46–5.48)** ^b^
Received information on importance of IFA	Yes	42	139	1.00	1.00
	No	71	68	0.29(0.18–.47)	**0.43(0.25–0.74)** ^b^
Morning sickness	No	160	95	0.65(0.35–1.18)	1.79(0.89–3.58)
	Yes	47	18	1.00	1.00
Knowledge on Anemia during Px.	Somehow knowledgeable	69	55	1.00	1.00
	less knowledgeable	44	152	0.25(0.14–0.43)	**0.23(0.14–0.38)** ^b^

Others^a^: Farmer, daily laborer

^b^Significant association at 95% confidence level

*Px* pregnancy, *COR* Crude Odds Ratio, *AOR* Adjusted Odds Ratio, *CI* Confidence Interval, *GA* Gestational Age, *ANC* Antenatal Care

These findings are supported by the FGDs and KIIS. Most of FGD’s participants reported that ANC is important for every mother to prevent different diseases and to have a healthy baby and visit should be started as early as possible. The majority of the respondents were able to describe the supplements and report the benefits of the supplements as to prevent anemia, replace blood and better fetal health. “*I believe that being followed by a doctor continuously is good to early detect any problem during pregnancy so that it could be managed as early as possible”*. ***(A 30-year old pregnant women having three children participated in FGD).*** All participants argued that should be taken once daily and continued throughout pregnancy.

## Discussion

In this study, 64.7%, with 95% CI (59.7% -70.0%), of pregnant women were found to be compliant with IFA. This finding is consistent with studies done in different regions of Nigeria (65.9%), Senegal (69%), India (64.7%) and Philippines (70%) [13, 14, 19, 20]. But it is a little bit lower as compared to the comparative study done in North Western Zone of Tigray, Ethiopia which revealed that 74.9% [15]. This finding is also inconsistent with the study done in Amhara region of Ethiopia which revealed that 20.4% adherence rate [20]. The probable reason may be their difference in living situation and the time gap between studies and study subjects. The reported level of adherence to IFA (64%) might have been overestimated because the measurements are via self-reporting method [15].

The number of ANC visits has a significant effect on adherence with IFA supplementation. ANC is a crucial channel for supplementing iron and folic acid during pregnancy. Therefore, the observed relationship between the level of ANC service utilization and level of IFA utilization is as expected. This finding is consistent with studies conducted in North Western Zone of Tigray, Ethiopia (AOR = 3.784, 95% CI = 2.073–6.909) and Philippines (*R* = 250.233, P, 0.01) [15, 20]. The reason might be that health providers could help women during their ANC visits by discussing adherence to IFA supplement, encouraging them to take the tablet as prescribed, and educating them on health benefit of taking IFA supplement. This result might be linked to the fact that women who receive health education about adherence to iron and folic acid by the health professionals during their regular follow-up period.

This study revealed that women who are more or less knowledgeable on anemia were more likely to be adherent with IFA. This is consistent with studies done in Nigeria and Ethiopia [13, 15, 17, 20]. Pregnant women who received information about the importance of iron supplementation during pregnancy were more likely to be compliant than those who were not. This is consistent with studies done in India and Sudan [16, 19].

Findings from the qualitative study revealed that forgetting to take drugs and fear of side effects are among the common reasons for lack of adherence to IFA in this study. Health professionals in charge of ordering IFA during pregnancy should apply detail counseling about the possible mild side effects of the drug and convince them that these side effects are limited and self-managed. Regarding the issue how to minimize forgetfulness, women should be continuously counseled to use different mechanisms like correlating with natural occurrences, sunrise or sunset, lunch or dinner, toilet use or hand washing so on and so forth.

Beside to fear of side effects and forgetfulness to take the drugs, which are factors found to be significant barriers for poor adherence of IFA in this study, negative perception about the drug, like fear of fetal complications and fear of making delivery more difficult are another factors found by different researchers so far [15, 21–23].

### Limitation of the study

This study has its own limitations. One of the limitations of this paper is the adherence level of the iron and folic acid among the pregnant women is simply determined by a self-report mechanism (the women’s response). This might affect the actual adherence level of the target population. Not all potential barriers to IFA adherence had been investigated and the measurement of the knowledge level of respondents about IFA is somehow subjective.

## Conclusions

The magnitude of adherence to IFA supplementation during pregnancy is relatively low in the refugee camps. This points out that the WHO and FMOHE recommendations were not met despite the fact that IFA supplementation program during pregnancy is used to prevent iron deficiency anemia during pregnancy.

The study finds out that counseling on IFA supplementation, knowledge of women on anemia and number of ANC visit were factors significantly associated with adherence to IFA supplementation during pregnancy. Both the quantitative and the qualitative results support that there are many barriers to adherence of IFA among pregnant mother like forgetfulness, drug side effects, and fear of side effects were the leading underlying reasons among women with low adherence. It is therefore crucial that pregnant women in the refugee camps should be given more focus in creating awareness and improving the knowledge level of the refugees about the adherence of iron and folic acid by the government and other supporting agencies. Government and other stakeholders should continue promoting the advantages timely ANC follow up, improve the quality of counseling in ANC service delivery and creating awareness and knowledge about anemia during pregnancy and about the negative perception about IFA drugs.

To minimize or prevent forgetfulness to take the drug, health professionals should counsel the women to correlate taking the drugs with natural events, like sunrise or sun sent, and day-to-day activities, like eating lunch or dinner. However, applying calendars are using alarms might not be helpful for these population as most of them are not literate. Regarding fear of side effects, health professionals in charge of giving the IFA drugs should continuously counsel and aware to the respondents that side effects are minor with no or minimum health problem, and if major side effects occurred, they should go to health facilities for management.

## Additional files

Additional file 1: A questionnaire used for collecting the quantitative data. (DOCX 32 kb)

Additional file 2: A questionnaire used for collecting the qualitative data. (DOCX 19 kb)

Additional file 3: The minimal anonymized quantitative data used for analysis. (ZIP 17 kb)

## Abbreviations

ANC: Antenatal Care
AOR: Adjusted Odds Ratio
ARRA: Administration for Refugees and Returnees Affairs
COR: Crude Odds Ratio
FGD: Focused Group Discussion
FMOHE: Federal Ministry of Health of Ethiopia
IFA: Iron-Folic Acid
IRC: International Refugee Camp
KII: Key Informed Interview
WHO: World Health Organization
