# Supplementary material for: Magnitude and factors associated with adherence to Iron-folic acid supplementation among pregnant women in Eritrean refugee camps, northern Ethiopia
Source: BMC Pregnancy Childbirth. 2018 Apr 5;18:83. doi: 10.1186/s12884-018-1716-2 (PMC5887183; doi:10.1186/s12884-018-1716-2)
Supplement: Supplementary file 1 — A questionnaire used for collecting the quantitative data. (DOCX 32 kb) [file 12884_2018_1716_MOESM1_ESM.docx]

**English Version Questionnaire for quantitative data**

| **Section I: Information Form**  001. Questionnaire code___________  002. Name of the Camp__________________  003. Respondent’s Zone _________________  **Instruction: Circle the appropriate answer** provided and where applicable writes the required responses in the spaces provided. (to be filled by data collectors) | | | | | | |  |
| --- | --- | --- | --- | --- | --- | --- | --- |
| **Section 1: Demographic and Socio-economic information** | | | | | | |  |
| **No** | **Questions** | | **Choices/answers** | | **Skip** | |  |
| 101 | How old are you? | | --------------Age in years | |  | |  |
| 102 | What is your current marital status? | | 1. Single 2. Married 3. Divorced 4. Widowed | |  | |  |
| 103 | Religion | | 1. Orthodox 2. Muslim 3. Catholic 4. Protestant 5. Others (specify)____________ | |  | |  |
| 104 | What is the highest level of school you attended? | | 1. No education 2. Grade 1-4 3. Grade 5 - 8 4. Grade 9- 12 5. Above 12 grades 6. Other (Specify)_______ | |  | |  |
| 105 | What is your present occupation? | | 1. House wife 2. Government employee 3. Merchant 4. NGO employee 5. Daily laborer 6. Private employee 7. Farmer 8. Other (Specify)_______ | |  | |  |
| 106 | What is the highest level of school your husband attended? | | 1. No education 2. Grade 1-4 3. Grade 5 - 8 4. Grade 9- 12 5. Above 12 grades 6. Other (Specify)_______ | |  | |  |
| 107 | The total family size in the house hold | | ____________ | |  | |  |
| 108 | Do you have your own income? | | 1. Yes 2. No | | 201 | |  |
| 109 | In total what is the monthly income of your family? | | 1. ____________ Birr 2. I don’t know/Not sure | |  | |  |
| **Section 2 :Obstetrics and health service factors** | | | | | | |  |
| **No** | | **Questions** | | **Choices/answers** | | **Skip** | |
| 201 | | How many times you get pregnant? (Gravida) | | ­­­­­­­­­­­­­­­­­­­­­­­­­­­______________ | |  | |
| 202 | | How many births do you have? (Parity) | | ­­­­­­­­­­­­­­­­­­­­­­­­­­______________ | |  | |
| 203 | | How many months pregnant are you now? | | ___________Months | |  | |
| 204 | | Do you have ANC follow up for your current pregnancy before? | | 1. Yes 2. No | | Q301 | |
| 205 | | How many times did you receive antenatal care during this pregnancy? | | 1. One 2. Two 3. Three 4. Four 5. Other _______(Specify) the reason for ANC) | |  | |
| 206 | | At what gestational age do you start ANC visit? | | 1. ---------------Months 2. I don’t remember/Not sure | |  | |
| **Session 3: Adherence to antenatal iron supplementation** | | | | |  | |  |
| **No** | | **Questions** | | **Choices/answers** | | **Skip** | |
| 301 | | During this pregnancy, did anyone tell you about the importance of taking iron tablets in pregnancy?  *(DC: show the tablets)* | | 1. Yes 2. No | | Q303 | |
| 302 | | If yes who told you about its importance? | | 1. Midwives 2. Medical doctor 3. Health social workers 4. Other (Specify)_______ | |  | |
| 303 | | What is the importance of taking iron tablets during pregnancy? | | ______________________________________________________________________________________________________________________________ | |  | |
| 304 | | How frequently should a pregnant woman take iron tablets? | | 1. On daily basis 2. On weekly basis 3. If other (specify) _________________ | |  | |
| 305 | | During this pregnancy, were you given or did you buy any iron tablets?  *(DC: show the tablets)* | | 1. Yes 2. No | | Q316 | |
| 306 | | At what gestational age did you start taking? | | __________Month | |  | |
| 307 | | Where did you get the iron tablets?  *(DC: multiple answers are possible)* | | 1. ARRA health centers 2. Hospitals 3. Private clinics 4. Drug vendors 5. If other (specify) _________________ | |  | |
| 308 | | If you received the iron tablets from a health professional/HEW, did he/she provide you a proper advice or information about it? | | 1. Yes 2. No 3. Not sure/I don’t remember 4. I did not receive it from H. Prof/HEW | |  | |
| 309 | | If you received the iron tablets from a health professional/HEW, did he/she tell you how long it will be taken? | | 1. Yes 2. No 3. Not sure/I don’t remember 4. I did not receive it from H. Prof/HEW | |  | |
| 310 | | If you received the iron tablets from a health professional/HEW, did he/she tell you about the right dose of the supplement? | | 1. Yes 2. No 3. Not sure/I don’t remember 4. I did not receive it from H. Prof/HEW | |  | |
| 311 | | In the preceding two weeks, for how many days did you take the tablets?  *DC: if answer is not numeric, probe for approximate number of days)* | | ------------------days | |  | |
| 312 | | During the whole pregnancy, for how many days did you take the tablets?  *(DC: if answer is not numeric, probe for approximate number of days)* | | ------------------days | |  | |
| 313 | | How do you rate your compliance for the iron supplement? | | 1. I used to take it on daily bases 2. I used to miss 1 or 2 tablets per week 3. I used to miss many days per week 4. I used to miss for more than a solid week 5. I stopped taking the tablet 6. I did not take any 7. Not sure/I don’t remember 8. If other (specify) _________________ | | Q315 | |
| 314 | | What where your main reasons for stopping or for not taking the tablets regularly? | | 1. Forgetfulness 2. I did not know its importance well 3. Fear of side effects 4. Occurrence of side effects 5. Husband’s disapproval 6. Did not get adequate tables from health institutions 7. If other (specify) _________________ | |  | |
| 315 | | What side effect you experienced?  *(DC: Don’t read the choices)*  *(DC: Probe, any others?)* | | 1. Nausea 2. Black stools 3. Constipation 4. If other (specify) _________________ | |  | |
| 316 | | Do you have morning sickness? | | 1. Yes 2. No | |  | |
| 317 | | Do you have any medical illness | | 1. Yes 2. No | |  | |
| 318 | | If answer for question 317is yes what medical illness do you have? | | 1. TB 2. HIV/AIDS 3. PUD 4. Other (Specify)_______ | |  | |
| **Section 4: Knowledge about maternal anemia** | | | | | | |  |
| **No** | | **Questions** | | **Choices/answers** | | **Skip** | |
| 401 | | What do you think is the major cause of extreme fatigue and dizziness during pregnancy?  *(DC: Register it in the respondent’s word)* | | 1. _______________________ 2. I don’t know/Not sure | |  | |
| 402 | | Have you heard about anaemia? | | 1. Yes 2. No | | End | |
| 403 | | What was your source of information about anaemia?  *(DC: multiple answers are possible)* | | 1. Health social workers 2. Health professionals (MD, nurses, HOs….) 3. Family/Neighbors 4. Mass media (TV, radio or newspaper) 5. Pamphlet/poster/leaflets/booklets 6. If other (specify) _________________ | |  | |
| 404 | | What do you think are the major causes of anaemia in pregnancy?  *DC: multiple answers are possible)*  *(DC: probe for more responses)* | | 1. Dietary iron deficiency 2. Illnesses like malaria 3. Intestinal parasitosis 4. Too close or too many births 5. Failure to take iron supplements 6. Blood loss 7. If other (specify) _________________ | |  | |
| 405 | | Do you know any consequence of anaemia in pregnancy?  *DC: multiple answers are possible)*  *(DC: probe for more responses)* | | 1. Increased risk of maternal death 2. Increased risk of serious maternal illness 3. Increased risk of fetal death 4. Poor mental development of the baby 5. If other (specify) _________________ | |  | |
| 406 | | Do you know how to prevent anaemia during pregnancy?  *DC: multiple answers are possible)*  *(DC: Probe, any others?)* | | 1. Consumption of iron reach foods 2. Intake of iron tablets 3. If other (specify) _________________ | |  | |
| 407 | | What are the major food items rich in iron? | | ____________________________________  ____________________________________  ____________________________________  ____________________________________  ____________________________________ | |  | |
