# Supplementary material for: Magnitude and factors associated with adherence to Iron-folic acid supplementation among pregnant women in Eritrean refugee camps, northern Ethiopia
Source: BMC Pregnancy Childbirth. 2018 Apr 5;18:83. doi: 10.1186/s12884-018-1716-2 (PMC5887183; doi:10.1186/s12884-018-1716-2)
Supplement: Supplementary file 2 — A questionnaire used for collecting the qualitative data. (DOCX 19 kb) [file 12884_2018_1716_MOESM2_ESM.docx]

**English version tool for qualitative data (FGDs and KIIs)**

1. **FGD guideline for pregnant women attending ANC**

Date of discussion: _______________

Facilitator’s name: __________________________________________

Note taker’s name: __________________________________________

Discussion started: _______________

Discussion ended: _______________

Camp: _______________

Dear participants;

Good morning/afternoon. I thank you for coming here today. I am __________________ who is a member of a team conducting a study on magnitude and factors associated with compliance to IFA supplementation among pregnant women in your community. The study is being conducted in all camps in Shire by Mekdemariam Getachew from Aksum University, department of public health, post graduate program. The purpose of this study is to assess the magnitude of compliance and associated factors with IFA supplementation among shire refugee pregnant women. Your participation in the study is on voluntary bases; however, your honest response and active participation will help us in understanding the situation better and will eventually contribute in designing appropriate interventions. All the information gathered will be kept confidential and your name will never be linked with any of the information that you will provide. Your participation is greatly appreciated.

**Background information about the discussants:**

| **No** | **Code of the participant** | **Age** | **Educational status** | **Camp** |
| --- | --- | --- | --- | --- |
| 1 |  |  |  |  |
| 2 |  |  |  |  |
| 3 |  |  |  |  |
| 4 |  |  |  |  |
| 5 |  |  |  |  |
| 6 |  |  |  |  |
| 7 |  |  |  |  |
| 8 |  |  |  |  |
| 9 |  |  |  |  |
| 10 |  |  |  |  |
| 11 |  |  |  |  |
| 12 |  |  |  |  |
| 13 |  |  |  |  |

Should a healthy pregnant woman attend ANC? Why? If not what kind of women should get the service?

1. Do you think ANC helps during pregnancy? How important is ANC for pregnant women? Why?

- Probe: How do other women in the community feel about it?
- Probe: Extent of perceived sense of vulnerability for life threatening problems during pregnancy.

1. Assess the knowledge of the pregnant women towards anemia.

- Ask: What do you think is the major cause of extreme fatigue and dizziness during pregnancy?
- Ask: Do you know anemia? What are its manifestations?
- Ask: Can you list some of its consequences in pregnancy?
- Ask: What possible prevention methods do you know?

1. Did you receive any health or nutrition education during this pregnancy?

- Probe: Source, messages and setting of the education.

1. Have you heard about iron tablet (show the tablet) which is given by health professionals during pregnancy?

- Ask: Why is it given for?
- Ask: How do you feel about taking a tablet during pregnancy?
- Ask: Have you ever received any education/information about taking iron tablet during pregnancy?
- Ask: Do you know any positive or negative perception or attitude in your community pertaining to it?

1. During this pregnancy did you take such tablet?

- Ask: Who prescribed the tablet to you?
- Ask: During the very first time IFS was prescribed to you, what kind of information did you receive from the health care provider about it?
- Ask: where the information and advices offered by the health care provider to you convincing and persuasive enough to start taking the tablet?
- Ask: Have you been taking the supplement regularly on daily basis? What are the reasons for discontinuation or for not taking it regularly?

1. In your opinion, what should be improved in order to enhance adherence of iron?

- Ask: What should health care system do?
- Ask: What should the community do?

1. **In-depth interview guideline for ARRA Health Professionals**

Date of interview: _______________

Interviewer’s name: __________________________________________

Interview started: _______________

Interview ended: _______________

Dear participants;

Dear participants;

Good morning/afternoon. I thank you for coming here today. I am __________________ who is a member of a team conducting a study on magnitude and factors associated with compliance to IFA supplementation among pregnant women in Shire refugee camps. The study is being conducted in all camps in Shire by Mekdemariam Getachew from Aksum University, department of public health, post graduate program. The purpose of this study is to assess the magnitude of compliance and associated factors with IFA supplementation among shire refugee pregnant women. Your participation in the study is on voluntary bases; however, your honest response and active participation will help us in understanding the situation better and will eventually contribute in designing appropriate interventions. All the information gathered will be kept confidential and your name will never be linked with any of the information that you will provide. Your participation is greatly appreciated.

**Background information:**

Code of the participant: ________________________

Position: ________________________

Camp: _______________________

Age of the interviewee: _______________

Total years of service: _______________

Service years in the current position: _______________

Educational status: _______________

1. Assess the knowledge of the health workers towards anemia and iron supplementation.

- Ask: What are the main manifestations of anemia during pregnancy?
- Ask: Can you list some of its consequences in pregnancy?
- Ask: What are the possible prevention methods?
- Ask: What is the need of providing IFS during pregnancy?
- Ask: What are the existing national recommendations regarding the dose and duration of IFS during pregnancy?

1. Did you ever get any on-job training regarding to IFS for pregnant women? When was the recent time that you got the training? Who offered the training?
2. How do you provide IFS to the beneficiaries?

- Probe: Personnel involved in the provision of the service, mode of delivery (home vs institution based), cost of service, and number of tables/strips provided at a visit.

1. Beyond providing the tablets do you really offer any special information, advice or counseling to pregnant women regarding IFS?

- Probe: Sufficiency of information, reasons for not providing advice, the contents of the information.
- Ask: Would you please tell us what you usually inform them?

1. How do you describe the logistics system of IFS in your health center?

- Ask: How does your health institution get IFS stock?
- Probe: On responsible body for distribution and supply.
- Explore: On the regularity, frequency and adequacy of IFS.
- Explore: On the existence of agreed system for early identification of stock out.

1. In the last one year, was there any instance that you did not have adequate IFS supplement to provide to pregnant women? If there was such instance, how long did the stock out last? How do you cope with such challenge?
2. In the last one year, was there any instance that the stock for IFS was not replenished despite your demand? If yes what was the reason?
3. Do you have sufficient stock of IFS in your health institution now?

- Observe: The existing stock and register the available quantity, type, expiry date and storage situation.

1. How do you describe the demand of the pregnant women for IFS? If there is poor demand what are the underlying reasons?

- Explore: Lack of awareness, fear of side effects, misconceptions, any other reasons.

1. What positive or negative perception or attitude related to ANC and IFS exist in the community?
2. How do you evaluate the adherence of pregnant women to IFS? What mechanisms are in place to monitor the level of adherence?

- Ask: What are the possible reasons for poor adherence?

1. So far have you made any special effort to enhance the enrollment of pregnant women into ANC and IFS? Discuss.
2. Apart from the issues already mentioned, what major challenges/problems do you commonly encountered in the delivery of ANC and IFS to pregnant women?
3. What do you suggest/recommend in order to improve the coverage and compliance to IFS?
